# Investigating the Effects of Mixing Dynamics on Twin-Screw Granule Quality Attributes via the Development of a Physics-Based Process Map

**DOI:** 10.3390/pharmaceutics16040456

**Published:** 2024-03-25

**Authors:** Lalith Kotamarthy, Subhodh Karkala, Ashley Dan, Andrés D. Román-Ospino, Rohit Ramachandran

**Affiliations:** Department of Chemical & Biochemical Engineering, Rutgers University, 98 Brett Road, Piscataway, NJ 08854, USA; lalith.kotamarthy@gmail.com (L.K.);

**Keywords:** twin-screw granulation, mixing, granule growth mechanisms, physics-based process map, continuous manufacturing

## Abstract

Twin-screw granulation (TSG) is an emerging continuous wet granulation technique that has not been widely applied in the industry due to a poor mechanistic understanding of the process. This study focuses on improving this mechanistic understanding by analyzing the effects of the mixing dynamics on the granule quality attributes (PSD, content uniformity, and microstructure). Mixing is an important dynamic process that simultaneously occurs along with the granulation rate mechanisms during the wet granulation process. An improved mechanistic understanding was achieved by identifying and quantifying the physically relevant intermediate parameters that affect the mixing dynamics in TSG, and then their effects on the granule attributes were analyzed by investigating their effects on the granulation rate mechanisms. The fill level, granule liquid saturation, extent of nucleation, and powder wettability were found to be the key physically relevant intermediate parameters that affect the mixing inside the twin-screw granulator. An improved geometrical model for the fill level was developed and validated against existing experimental data. Finally, a process map was developed to depict the effects of mixing on the temporal and spatial evolution of the materials inside the twin-screw granulator. This process map illustrates the mechanism of nucleation and the growth of the granules based on the fundamental material properties of the primary powders (solubility and wettability), liquid binders (viscosity), and mixing dynamics present in the system. Furthermore, it was shown that the process map can be used to predict the granule product quality based on the granule growth mechanism.

## 1. Introduction

Wet granulation is a particle size enlargement process in which fine powders agglomerate in the presence of a non-toxic liquid binder [1]. Wet granulation is governed by three important rate mechanisms: (i) wetting and nucleation, (ii) consolidation and growth, and (iii) breakage and attrition [2]. Due to its numerous advantages, this process is employed across many industries such as in food, minerals, detergents, pharmaceuticals, etc. [3,4]. The pharmaceutical industry often utilizes the wet granulation technique due to its versatility in manufacturing a wide range of formulated products. Over the past decade, there has been a shift toward continuous powder manufacturing, in part due to the introduction of the quality-by-design (QbD) and process analytical technology (PAT) guidance by the Food and Drug Administration (FDA) in 2004 [5]. Twin-screw granulation (TSG) is a popular continuous wet granulation technique that was first introduced in the late 1980s and was later patented in 2002 [6]. TSG provides numerous advantages such as an improved production efficiency, mitigation of scale-up issues, improved product quality, and versatile formulation processability due to its continuous manufacturing capabilities and modular design [7,8].

Studies have shown that the particle size distribution, granule content uniformity, and granule microstructure are the most important granule quality attributes, and these three properties together affect the downstream flow properties and tablet quality properties [7,9,10]. A poor granule content uniformity (defined as the measure of the active ingredient distribution across different size classes), which is characterized by the irregular distribution of active ingredients within different size fractions, can result in non-uniform tablets and irregular tablet dissolution. A granule microstructure encompasses several properties such as porosity, surface morphology, component content uniformity, pore size distribution, open-to-closed pore ratio, etc. All these factors together affect the flow, strength, content uniformity, and stability of the granules [3,11,12,13] and also affect the critical quality attributes of downstream final dosage forms like tablets and capsules [7,14,15,16]. Studies have shown that variance in the porosity of the granules significantly affects the strength and dissolution of tablets [8,15,17,18,19].

Few studies have focused on understanding the effects of the TSG input parameters on the granule microstructure. A few researchers studied the mechanism of granule formation along the barrel length of a twin-screw granulator (barrel diameter = 16 mm, L/D = 25) and found that conveying elements alone created very porous granules (nuclei-like), and in the presence of kneading elements, the density of the granules increased with the increase in kneading elements to a certain point before breakage was observed [12,20]. Another study observed that kneading elements led to the formation of granules that had their pores distributed towards the surface of the granules, indicating a dense core. On the contrary, the granules formed due to distributive mixing elements were more porous, and the pore network extended throughout the granule uniformly [14].

Even fewer studies have attempted to understand the effects of different TSG parameters on granule content uniformity across different size fractions. Yu et al. [21] found that an increase in the liquid content and an increase in the number of kneading elements improved the content uniformity of the granules due to improved nucleation and improved liquid–solid distribution, respectively. Mundozah et al. [12] observed that viscosity dampened the mixing conditions in the zone and led to the formation of content non-uniform granules, especially in the presence of kneading and tooth-mixing zones.

These studies gave useful insights into what occurs inside a twin-screw granulator but did not highlight the fundamental factors/mechanisms that affect the granule microstructure and granule content uniformity. Some of these studies used single-component formulation, whereas some others did not vary the process, screw, or/and material properties, which led to a lack of understanding of the complex interactions of these parameters that can occur in the system. Moreover, many of these studies hinted at mixing as the critical factor affecting the granule quality attributes, but they failed to analyze and link the effects of mixing dynamics or other fundamental factors to the granule microstructure or granule content uniformity. Therefore, it is essential to understand the effects of mixing dynamics on the final granule quality attributes by incorporating the interaction effects among process parameters, equipment parameters, and material properties. To accomplish this meaningful, physics-based intermediate parameters encompassing the effects of the process, screw, and material properties need to be identified, estimated, and analyzed. This will help improve the mechanistic understanding and prediction of the granule growth behavior, which will eventually improve the understanding and prediction of the granule quality attributes in TSG.

### Objectives

This study aims to identify the fundamental parameters that affect the mixing dynamics, and as a result, the physics occurring in TSG, to help improve the predictability of granule quality attributes. To achieve this objective, this study will build on the understanding obtained in one of the previous studies performed by the authors, and in that study, the authors focused on understanding the effects of the material properties on the mixing dynamics, and consequently, on granule product quality. Therefore, in this current study, the focus will be on the process and equipment parameters, specifically the screw speed, number of kneading elements, and the distance between the liquid addition zone and kneading zone. The effects of the process and equipment parameters on granule quality attributes such as the PSD, content uniformity, and microstructure will be analyzed with the aid of the axial dispersion coefficient (mixing metric). To refine this analysis, the physically relevant intermediate parameters of TSG were quantified and utilized. Eventually, this investigation will be used to identify the fundamental parameters affecting the mixing dynamics, and their effects on the granulation growth dynamics occurring in the system will be elucidated. Finally, a process map depicting the evolution of a granule in a twin-screw granulator in terms of space and time will be developed, linking the input parameters to the output quality attributes via mixing and granulation rate mechanisms.

## 2. Materials and Methods

### 2.1. Materials

In this study, acetaminophen (APAP) manufactured by Mallinckrodt Pharmaceuticals in Raleigh, NC, USA, was utilized as the active pharmaceutical ingredient (API). Microcrystalline cellulose (MCC; Avicel PH 102 grade), supplied by FMC Corporation in Philadelphia, PA, USA, served as the excipient. The material properties of the raw materials are listed in Table 1. Distilled water was used as the liquid binder for all the experiments conducted in this study.

### 2.2. Granule Preparation

Acetaminophen (APAP) of a dense-powder grade and microcrystalline cellulose (MCC) were mixed together in a V-blender from Glatt Air Techniques Inc. in Ramsey, NJ, USA. The blending time was set to 15 min, and the composition comprised 15% API and 85% excipient. This pre-blend was then added to the twin-screw granulator using the MT-12 gravimetric loss-in-weight twin-screw feeder (K-Tron Soder in Niederlenz, Switzerland). The TSG experiments were conducted employing an 11 mm co-rotating twin-screw granulator from Thermo Fisher Scientific in Dartford, UK. This twin-screw granulator had a horizontal split barrel with a length-to-diameter (L/D) ratio of 40:1. Water was used as the liquid binder, and a stagger angle of negative sixty degrees was employed in all experiments. Zone 2 of the twin-screw granulator was designated as the powder addition zone, while zone 3 was designated as the liquid addition zone. The granules were collected during the steady-state operation of the twin-screw granulator, which was identified by observing the stable torque values. The obtained wet granules were dried in a convection oven at 40 °C until the moisture content reached below 4% [7,22]. The subsequent characterization of the granules was performed on the dried granules.

As mentioned earlier, this study focuses on understanding the effects of process and equipment parameters on the mixing dynamics, and in turn, the granule product quality. Specifically, this study will concentrate on the screw speed, the number of kneading elements, and the position of the kneading zone relative to the liquid addition zone, as these are some of the most important parameters that affect granulation in TSG. To gain an understanding of these parameters, the experiments were designed such that the input parameters were varied one factor at a time (OFAT), leading to a total of ten experiments. The variation range of each factor is shown in Table 2. The use of such an experimental design to gain of a mechanistic understanding of wet granulation processes has been commonly used in literature [12,14,23,24,25,26,27,28].

For all the experiments, the feed rate was kept constant at 0.8 kg/h, and water was used as the granulation liquid. The kneading elements used had a thickness of D/6 and were arranged with a stagger angle of negative 60 degrees.

### 2.3. Residence Time Distribution

As explained in the previous study by the authors [29], the selection of appropriate materials is crucial for conducting residence time distribution (RTD) experiments. It is important that the flow properties of the tracer material and the components in the formulation, particularly the active pharmaceutical ingredient (API), are similar [30]. In a wet granulator, which is a multi-phase system, the solubility and wettability of the powders are two key factors influencing the flow properties of the system. In this study, ibuprofen was chosen as the tracer material, because it exhibits similar wettability characteristics to dense APAP [3,29]. A quantity of 200 mg of ibuprofen was introduced as a pulse to capture the RTD, and this procedure was repeated three times for each experiment. To analyze the residence time distribution (RTD) of the experimental runs in this study, the Raman spectroscopy technique was employed. The Raman instrument utilized in this study was the HyperFlux™ Raman spectrometer from Tornado Spectral Systems, located in Ontario, Canada. The Raman probe, known as the Hudson probe, was positioned to collect continuous data by averaging 5 exposures of 100 ms each, resulting in the acquisition of 2 spectra per second. The collected spectral data was then uploaded to SIMCA 16 (Goettingen, Germany). Subsequently, a partial least squares (PLS) calibration model was generated using five calibration runs, which covered ibuprofen concentrations ranging from 0.0 to 9.0 *w*/*w*. In total, 720 calibration spectra were obtained. The data acquisition process commenced after attaining a steady state and lasted for 6 min. The Raman spectra were subjected to pretreatment within the spectral range of 200–3300 cm^−1^. The pretreatment of the data and determination of the number of latent variables were optimized by evaluating the root mean square error of cross-validation. However, these values were solely utilized to establish the configuration of the model, and the final model was independently tested in a separate run. The model achieved accurate predictions by employing the standard normal variate (SNV) for the data pretreatment and utilizing 3 latent variables. A similar process was used by the authors in their previous study to collect and analyze RTD data [29].

### 2.4. Analytical Characterization of Granules

#### 2.4.1. Particle Size Distribution

In this study, the particle size distribution (PSD) is defined on a mass fraction basis. The particle size distribution was determined through the process of sieve analysis. This involved using a set of sieves with different mesh sizes, including pan, 90 μm, 180 μm, 250 μm, 355 μm, 500 μm, 710 μm, 850 μm, 1000 μm, 1400 μm, 2000 μm, 2380 μm, 3350 μm, 4000 μm, and 4750 μm. The Endecotts Octagon 2000 sieve shaker, manufactured by Endecotts in London, UK, was employed for the analysis, with an amplitude setting of 8. This experiment was conducted by dividing the sieves into two fractions: coarse and fine. The coarse fraction comprised particles ranging from 4.75 mm to 1 mm, while the sub-1 mm particles were categorized as the fine fraction. The coarse analysis took approximately 5 min to complete, whereas the fine analysis required about 10 min. The important metrics of PSD (D10, D50, and D90) were also calculated and analyzed. D10 (μm) is defined as the particle size below which 10% of the mass of the granules exists. Similarly, D50 (μm) and D90 (μm) are defined based on 50% and 90% of the masses of the granules, respectively. This method was employed by the authors in their previous studies [7,17,22,29].

#### 2.4.2. Content Uniformity

The content uniformity of the granules was evaluated using UV–vis spectroscopy (Specord 205 BU, Analytik Jena, Germany). Methanol was chosen as the diluent for measuring the acetaminophen (APAP) content, because APAP’s solubility in methanol is approximately 25 times greater than in water [31]. The absorbance of pure acetaminophen dissolved in methanol (histological grade) was found to be the highest at 248 nm. Hence, a calibration chart was created by measuring the absorbances at 248 nm of known concentrations of pure acetaminophen dissolved in methanol.

For the calibration curve, the concentration of pure acetaminophen (APAP) in methanol ranged from 0.0025 μg/mL to 0.02 μg/mL. A linear calibration curve was constructed within this concentration range, and it exhibited a strong correlation with an R2 value of 0.9974. To analyze the content uniformity of the granules, 200 mg of granules were selected from each size fraction or sieve cut. These granules were crushed into fine powders to facilitate a faster dissolution. This method was employed to closely resemble the actual content uniformity analysis conducted during the calibration method development. The 200 mg of crushed granules were then dissolved in 50 mL of methanol, creating a stock solution. The stock solution was subjected to sonication for 1 h to ensure the complete dissolution of APAP from the granules. Subsequently, the stock solution was diluted 60 times to achieve a concentration that fell within the range covered by the calibration curve. The authors have adopted this method from their previous study [29].

Analyzing the quantitative aspects of the product quality is crucial for enhancing the understanding of a process at a mechanistic level. In the context of content uniformity, the shape of the curve obtained as a function of the particle size provides qualitative information regarding the presence of sub-potent or super-potent fines. However, comparing the content uniformities across different experiments and drawing conclusions can be challenging. Furthermore, it is possible to observe sub-potency or super-potency of fines at various particle sizes in different experiments, making it even more difficult to make comparisons. Therefore, it is important to quantify the content uniformity to comprehend the impacts of different input parameters. Thiel and Nguyen [32] introduced a metric known as the de-mixing potential, which represents the coefficient of variation of the minor component (API) in different-sized fractions. This de-mixing potential is mathematically defined by Equation (1).

(1) $$DP\left(\%\right)=\frac{100}{\bar{x}}\sqrt{\sum w{\left(x-\bar{x}\right)}^{2}}$$

The de-mixing potential (*DP*) is a metric that quantifies the content uniformity of granules. It is calculated based on the average API content ($\bar{x}$) across all the granules, which typically represents the intended API content. The API content in the granules of a specific sieve cut (x) and the mass fraction of that sieve cut (w) are also considered in the calculation. By incorporating the API content and mass fraction of each sieve cut, the de-mixing potential becomes a reliable quantitative metric for evaluating the content uniformity. It describes the extent to which the API content deviates from the mean value. In general, a lower value of DP indicates better uniformity of the API across different size classes. This suggests a reduced deviation, indicating a more consistent API content throughout the granule samples.

#### 2.4.3. Granule Microstructure

In this study, the microstructure of the granules was analyzed using micro-computed tomography (micro-CT) equipment, specifically the Skyscan 1272 from Microphotonics/Bruker (Billerica, MA, USA). The micro-CT equipment operates by generating an X-ray beam through the projection of an electron beam onto a brass target. This X-ray beam then passes through the sample, which is positioned on a rotation stage, and the resulting transmitted image is captured using a sensitive X-ray detector. To ensure consistency and uniformity in the analysis of the microstructure, granules within the size range of 710–850 μm were selected from each batch. These granules were carefully mounted onto narrow plastic tubes, which were subsequently placed on the rotation stage of the micro-CT equipment. To prevent any dislodgement during the rotation process, superglue was utilized to secure the granules onto the plastic tubes.

The micro-CT analysis utilized an X-ray source voltage of 40 kV, which generated an amperage of 200 μA. Each granule underwent a 180° scan, and the frame averaging was set at 3, indicating the number of scans per scan step. The analysis was performed without the use of a filter, and the scan step was 0.3°. The resulting images had a pixel count of 2504 × 1920, leading to a resolution of 2.1 μm per pixel.

To prepare the raw stack images obtained from the micro-CT equipment for post-scan analysis, they were first cropped to remove most of the empty background and the superglue attached to the bottom surface of the granule. The resulting cropped image was then processed using Vivoquant 3.0 software to generate final binarized microstructure images of the granules. Binarization involved segmenting the image based on the CT densities of the different components, namely APAP and MCC.

To ensure uniformity across all granules from different experiments, the CT density analysis was performed on one of the pure components. Specifically, pure APAP granules were manufactured and subjected to a micro-CT analysis within the size range of 700–850 μm to obtain the CT density of APAP. The manufacturing of APAP granules was necessary to obtain particles of the minimum size required for the micro-CT analysis. The analysis revealed that the CT densities of APAP granules mostly fell within the range of 1000–1400. Additionally, the CT density of the background, which served as a reference, was known to be between −1000 and 0. The authors have adopted this method from their previous study [29].

### 2.5. Mixing Analysis

Analyzing and quantifying the mixing process enhances our understanding of the mixing dynamics in systems like TSG (twin-screw granulation). In this study, the axial dispersion coefficient is utilized as a quantitative metric to measure mixing, as axial dispersion is identified as the primary factor contributing to mixing in TSG. The experimental method for determining and analyzing the axial dispersion coefficient in this study is adopted from the approach proposed by Kotamarthy et al. [4]. This method is based on the dispersion model, which is mathematically described in Equation (2). According to this method, the variance of the RTD curve is obtained first, and the mean-centered variance (MCV) is calculated by dividing the variance by the square of the mean residence time (MRT). Then, the Peclet number is obtained from the MCV using the correlation shown in Equation (3), and this equation holds based on the assumption that TSG is an open–open system. From the Peclet number, the axial dispersion coefficient is obtained using the correlation shown in Equation (4).

(2) $$\frac{\partial C}{\partial t}=D\frac{\partial^{2}C}{\partial z^{2}}-\frac{\partial \left(UC\right)}{\partial z}$$

In Equation (2), *C* is the concentration, *D* is the dispersion coefficient, *z* is the direction of the flow, *U* is the velocity of the stream in the direction of the flow, and *t* is the time.

(3) $$\frac{\sigma^{2}}{{t_{m}}^{2}}=\frac{2Pe+8}{{\left(Pe+2\right)}^{2}}$$

In Equation (3), σ is the variance, $t_{m}$ is the mean residence time, and *Pe* is the Peclet number.

(4) $$Pe=\frac{Rate\;of\;axial\;transport\;by\;convection}{Rate\;of\;axial\;transport\;by\;dispersion}=\frac{U_{s}*l}{D_{a}}$$

In this context, the Peclet number (Pe) is determined based on several parameters. These include the superficial velocity ($U_{s}$), which represents the highest achievable velocity of a powder–liquid system within a system; and the characteristic length (l), which defines the length of the system that governs the fluid’s mechanical behavior. Furthermore, the axial dispersion coefficient ($D_{a}$) is also taken into account.

To analyze the superficial velocity in this study, the correlation presented in Equation (5) was employed. The characteristic length (l) is specifically determined as the length of the system that most significantly influences the fluid’s mechanical behavior. Kotamarthy et al. [4] demonstrated that the length of the kneading zone best represents the characteristic length of the twin-screw granulator, as this zone exhibits the maximum impact on the fluid’s mechanical behavior within the system.

(5) $$U_{s}\left(cm/s\right)=\frac{active\;length\;of\;the\;granulator\;(cm)}{t_{min,\;avg}^{*}\;(s)}$$

For this study, the active length of the granulator, spanning from zone 2 to the exit, was determined to be 35.5 cm. Additionally, $t_{min,\;avg}^{*}$ represents the average minimum residence time measured across the repetitions of the residence time distribution (RTD) pulses for each experiment.

### 2.6. Mathematical Model Development for Physically Relevant Intermediate Parameters

To improve the mechanistic understanding of the effects of intermediate parameters on mixing and the consequent granulation growth dynamics, a quantification of the intermediate parameters was performed. This quantification is especially useful for the fill level and energy supplied to the powders, as these parameters are affected by a complex interplay between different input parameters. Moreover, these intermediate parameters are representative of the shear/energy applied to the particles in the system.

#### 2.6.1. Estimation of Fill Level

As mentioned earlier, the fill level is defined as the ratio of space occupied by the powders to the maximum space available. Mundozah et al. [27] provided a geometrical model for the estimation of the twin-screw granulator that is fairly mechanistic, but this model does not take into account the effect of the stagger angle in the twin-screw granulator. The previous literature showed that the stagger angle has a significant effect on the fill level, and thus, on the quality of the granules formed [33,34]. Hence, it is important to incorporate the effect of the stagger angle into the estimation of the fill level in the twin-screw granulator. Thus, a new model incorporating the effect of the stagger angle was developed in this study, and this model was built off the existing model developed by Mundozah et al. [27]. The following equation scheme was used to estimate the fill level:

(6) $$space\;occupied\;by\;powders\;\left(g\right)=FR*\frac{V_{free}}{V_{max}}*MRT$$

(7) $$\max space\;available\;to\;occupy\;\left(g\right)=\rho_{bulk}*V_{max}$$

(8) $$fill\;level\left(\emptyset \right)=\frac{space\;occupied\;by\;powders}{\max space\;available\;to\;occupy}=\frac{FR*V_{free}*MRT}{\rho_{bulk}*V_{max}^{2}}$$

Here, FR is the powder feed rate, MRT is the mean residence time, $V_{free}$ is the free volume available for powders to occupy, $V_{max}$ is the maximum volume available, and $\rho_{bulk}$ is the bulk density of the powders. Though there is granule formation across the barrel, it is safe to assume that the average bulk density is approximately equal to the powder bulk density [23,27,35]. $V_{free}$ and $V_{max}$ are estimated using the following equation sequence:

(9) $$V_{max}=L_{eff}*A_{T}$$

(10) $$A_{T}=2*A_{C}-A_{E}$$

(11) $$V_{free}=V_{max}-V_{shaft}-V_{element}$$

(12) $$V_{shaft}=L_{eff}*A_{s}$$

(13) $$V_{element}=V_{conv}+V_{kneading}+V_{DME}$$

(14) $$V_{kneading}=2*kneading\;zone\;length*A_{blocked\;cross\;section}$$

(15) $$A_{blocked\;cross\;section}=2*\frac{1}{2}*R^{2}*\left(SA\right)$$

where $L_{eff}$ is the length of the barrel used for granulation, $A_{T}$ is the total available cross-sectional area, $A_{C}$ is the cross-sectional area of the cylinder formed by a single barrel, $\;A_{E}$ is the cross-sectional area of the overlapped eclipse between the two barrels, $V_{shaft}$ is the volume of the shaft, $A_{s}$ is the cross-sectional area of the shaft, $V_{element}$ is the total volume occupied by elements, $V_{conv}$ is the volume occupied by conveying elements, $V_{kneading}$ is the volume occupied by conveying elements, $V_{DME}$ is the volume occupied by DMEs, $A_{blockedcrosssection}$ is the cross-sectional area of the blockage provided by kneading elements, R is the radius of the kneading element, and SA is the stagger angle between the kneading element. $A_{blockedcrosssection}$ accounts for the blocked space created by the kneading discs arranged at a particular stagger angle. It was previously observed in the literature that increasing the stagger angle creates an additional blockage to the flow of powders, leading to a decreased conveying capacity and an increase in the powder fill level [33,36,37]. This was accounted for by calculating the two minor sector areas (comprising of the stagger angle) formed between two adjacent kneading discs. The volume of conveying elements and DMEs was obtained experimentally using a volume displacement technique.

$V_{conv}$ and $V_{DME}$ are estimated using the volume displacement method. $A_{s}$ can be easily calculated, as it is in the shape of a regular hexagon.

#### 2.6.2. Energy Supplied to the Powders

The energy supplied to the powders provides an estimate of the shear applied to the powders. As a consequence of the shear applied, the particles in the system undergo collisions, and therefore, the energy supplied to the powders also gives us an estimate of collision frequency and collision kinetic energy [38]. Several researchers in the past have tried to use or highlight the importance of specific mechanical energy (SME) as a surrogate parameter to analyze the energy supplied to the system [6,27,35,39]. This parameter itself was adopted from twin-screw extrusion, where extensive studies have been performed to understand the effect of this parameter as an important physically relevant intermediate parameter [40,41]. Quantitatively, specific mechanical energy (SME) can be determined by using Equation (16), where τ is the torque supplied to the system, ω is the screw speed in radians per second, and FR is the powder feed rate of the system.

(16) $$SME=\frac{\tau \omega }{FR}$$

One major drawback of this measurement concerning the twin-screw granulator is that the effect of the screw configuration (especially kneading elements) is not explicit. It is expected that the effect of the screw configuration would be implicit within the torque value, but it has been shown in the literature that the screw configuration affects fundamental properties of twin-screw granulation such as the shear, flow and mixing patterns, fundamental rate mechanisms, etc. [4,34,42]. So, for such an important parameter, it is a little presumptuous to implicitly conclude that the effect of torque is not sufficient. For the wide range of screw configurations that are possible, it is easy to visualize a scenario where two different screw configurations can lead to similar torque values (at a similar ratio of screw speed and feed rate), but the resultant granule properties could be very different due to a difference in the fundamental dynamic properties such as the shear, flow and mixing patterns, and rate mechanism, especially since the torque is affected by a complicated interplay between the viscous nature of the fluid and the flowability of the system. For example, Mundozah et al. [12] showed that adding a distributive conveying element after the wetting and nucleation zone facilitated breakage due to an increased shear and reduced internal volume, which, in turn, increased the barrel fill level and collision rate of the particles. This resulted in granules having an increased porosity with an improved content uniformity and slightly increased fine fraction. However, it was observed that the torque of the system was not affected significantly due to the introduction of the distributive conveying element. Moreover, when the viscosity of the binder was increased, there was no change in the torque, but the granule quality attributes were significantly affected, while the L/S ratio, screw speed, and feed rate of the system were kept constant. A similar observation was made by Dhenge et al. [39], when they varied the viscosity of the binder with the same screw configuration using the same process parameters. This conclusively shows that the SME alone is not a sufficient parameter to estimate the energy supplied to the powders, and consequently, is not sufficient to study collisions and flow patterns. Another significant parameter that affects the dynamics in the twin-screw granulator is the fill level, as seen earlier. Both Dhenge et al. [39] and Mundozah et al. [12] reported that though the torque of the system did not vary, the fill level and the flow patterns of the system varied with increased binder viscosities and the introduction of new elements such as kneading elements, distributive mixing elements, etc., respectively. Furthermore, Mendez et al. studied the stresses occurring inside the granulator by introducing microencapsulated stress sensors along with the powders into the twin-screw granulator. They reported that the stresses occurring in the granulator increase with the fill level to a certain extent and then decrease again. But the trend of specific mechanical energy as a function of the fill level was decreasing, according to their report. This indicates that the fill level has to be incorporated into the energy equation to improve the mechanistic understanding of the collisions occurring in the system.

As established, the missing piece of information in the energy estimation metric in TSG is the fill level. The specific mechanical energy calculates the energy as a function of the feed rate, but to understand the effect of the energy provided on the collisions and the dynamics inside the twin-screw granulator barrel, a calculation of the energy as a function of the particles existing in the barrel must be performed, which is nothing but the holdup. Hence, in this study, the estimation of the energy provided to the system was carried out by using Equation (17).

(17) $$E_{s}=\frac{\tau \omega }{\varphi }$$

(18) $$\varphi \;(holdup)=FR*MRT*\frac{V_{free}}{V_{max}}$$

Here, $E_{s}$ is the energy supplied to the powders per unit time per unit mass, τ is the torque of the system, ω is the screw speed in radians per second, φ is the holdup inside the barrel, FR is the powder feed rate introduced, MRT is the mean residence time, $V_{free}$ is the free volume existing in the barrel, and $V_{max}$ is the maximum space available for occupation. $V_{free}$ explicitly takes into account the effect of the screw configuration, and between the torque and mean residence time, fundamental dynamics such as the shear, flow pattern, and rate dynamics are taken into account. The energy supplied per unit mass per unit time ($E_{s}$) is an important physics-based parameter governing the mixing dynamics, according to turbulent mixing principles [4]. This further validates the use of $E_{s}$ as a physics-based intermediate parameter to understand collisions inside the twin-screw granulator.

The average total energy ($E_{avg}$) imparted to a unit mass of material is another important metric to understand the shear applied to the powders in a twin-screw granulator. The average total energy ($E_{avg}$) imparted would give an estimate of the energy subjected to a unit mass of material during its conversion from powder to granule in a twin-screw granulator, which would essentially represent the average shear undergone by particles inside the twin-screw granulator. Mathematically, the average total energy ($E_{avg}$) is given by Equation (19) and has the units of J/g.

(19) $$E_{avg}=E_{s}*MRT$$

## 3. Results and Discussion

### 3.1. Verification of the Proposed Fill Level Model

**Figure 1:**
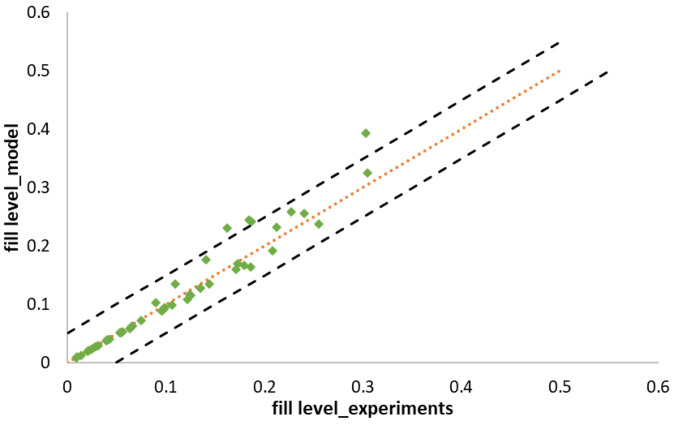
Fill level estimated vs. fill level reported in earlier studies. Orange dotted line (......) represents the x = y line, green dots (^◆^) represent the estimated fill level values, and black dotted lines (**- -**) represent the 5% error margins.

To verify the proposed fill level model, the fill level was calculated from the experimental data present in the literature and was compared to the results presented in those studies. The experimental data were chosen from Meier et.al. [43] and Mundozah et.al. [27]. Figure 1 shows the parity plots between the fill level estimated using the method proposed in this study and the reported values from the experimental data collected. It can be observed that the proposed model predicted the fill level values accurately. Only 4 values (out of 46 runs) fell outside the 5% error boundaries, and the mean absolute error was found to be equal to 0.00139.

Mundozah et al. studied the effects of varying liquid and mass flow rates using polymethyl methacrylate (PMMA) as the feed powder. They also studied the effects of varying screw elements for both lactose and PMMA as the feed material, and at each level of the above parameters, the screw speed was varied at four different levels, and the proposed model captured the effects of all these parameters on the fill level accurately.

### 3.2. Effect of Screw Speed

For this set of experiments, zone 4 was used as the granulation zone, and six kneading elements were employed in the granulation zone. An L/S ratio of 100% was used for these experiments.

**Figure 2:**
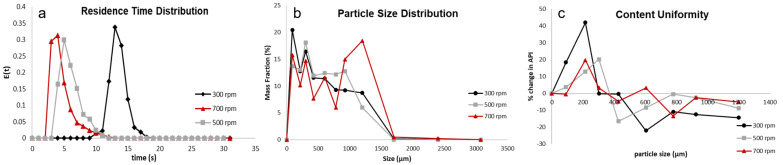
(**a**) Effect of screw speed on RTD, (**b**) effect of screw speed on PSD, and (**c**) effect of screw speed on content uniformity.

The screw speed affects the dispersion in a twin-screw granulator in a significant manner. From Figure 2a, it can be seen that with an increase in the screw speed, the mixing (width of the RTD curve) efficiency and the dispersion coefficient increase. The authors observed this phenomenon in an earlier paper [4] and proposed that an increase in the screw speed leads to an increase in the velocity differential amongst the streams in the radial direction, and this leads to different streams traveling with different velocities. The creation of new streams with different velocities leads to an increase in the encounters/collisions of particles with different temporal histories, leading to an increase in the axial dispersion coefficient and mixing efficiency.

**Figure 3:**
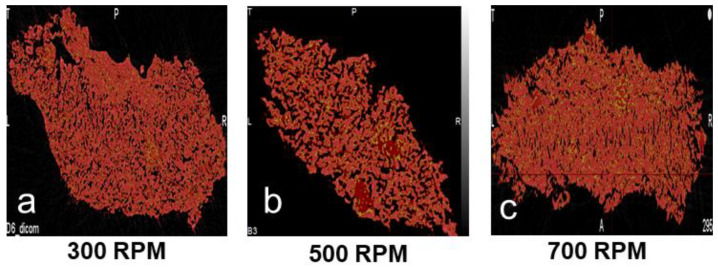
Effect of screw speed on the granule microstructure: (**a**) 300 rpm, (**b**) 500 rpm, and (**c**) 700 rpm.

An increase in the axial dispersion coefficient leads to an improvement in axial mixing, and consequently, an improvement in the liquid–solid distribution [34]. The improvement in the liquid–solid distribution is either achieved via an increase in the extent of consolidation or an increase in breakage or both. This leads to increased availability of surface liquid, leading to the promotion of granule growth due to improved coalescence. This can be observed from the PSD of the granules obtained at the three different screw speeds (Figure 2b). From Figure 2b, it can be observed that with the initial increase in screw speed from 300 rpm to 500 rpm, there was an increase in the formation of larger granules, and the width of the distribution decreased, indicating an improved and better growth dynamic due to improved coalescence. With a further increase in the screw speed, there was a clear increase in the formation of larger granules and a decrease in the fraction of smaller granules produced, but the width of the PSD increased, and the shape of the PSD obtained was irregular. An increase in the screw speed from 500 rpm to 700 rpm only affected the ratio between the drop penetration time and encounter time ($D_{t}/E_{t}$) narrowly: the value increased from 1.07 to 1.49. It was observed in a previous study that at low $D_{t}/E_{t}$ values (close to 1), the nuclei formation for this type of formulation occurs via immersion nucleation [29], which eventually leads to the layering-dominant growth of the granules. Granules formed by the layering-dominant growth mechanism are known to be brittle and susceptible to breakage [44]. When the screw speed increased to 700 rpm from 500 rpm, the energy supplied to the powders per unit time per unit mass ($E_{s}$) increased significantly, greater than three times (Table 3). This increase in the energy could have led to an increase in the collision energy and breakage of the weak immersion nuclei and layering-dominant granules, which, in turn, produced fresh faces of granules with surface liquid, consequently promoting granule growth. This phenomenon of extensive breakage followed by growth (breakage-dominant growth) resulted in the irregular shape of the PSD observed at 700 rpm (Figure 3b). The promotion in granule growth due to improved coalescence could be the result of increased viscous dissipation (Type 1 coalescence), increased deformation (Type 2 coalescence), or both [45,46]. This increase in the apparent surface liquid of the granules leading to a promotion in the granule growth can occur irrespective of the type of growth occurring in the system, “layering-dominant” or “viscous-dominant”, and hence, the type of nucleation occurring in the system (immersion nucleation or incomplete nucleation) [38].

It has been established that with an increase in the screw speed, axial dispersion and mixing in the twin-screw granulator improved. This improvement in mixing resulted in an improved solid–liquid distribution in the kneading zone, either through an increase in the extent of consolidation or an increase in the breakage. In the case of an increase in the extent of consolidation, the liquid is squeezed to the surface due to the compaction of the granule [2,47,48], leading to an increase in the surface wetness of the granule. In the event of the breakage of a granule, newer wet surfaces of the granules are exposed [34,49,50]. Moreover, the breakage of granules generally leads to an increase in the surface area-to-volume ratio, thereby increasing the apparent value of the surface wetness. This increase in the surface wet area promotes granule growth and solid component exchange. The growth can be either via coalescence between two surface wet granules or via the layering of smaller, finer particles onto the larger surface-wet granule. But in either case, the exchange of solid components between different size classes is enhanced, resulting in an improved content uniformity as shown in Figure 2c. This observation was validated by the trend of de-mixing the potential of the granules (Table 3), which decreased with an increase in the screw speed.

It can be observed from Figure 3 that with an initial increase in the screw speed, the granule porosity increases and subsequently decreases with a further increase in the screw speed. From Table 3, it can be observed that with an increase in the screw speed from 300 rpm to 500 rpm, the fill level of the system dropped by more than 50%, and the energy supplied to the granules per unit time per unit mass ($E_{s}$) increased. From the granule growth behavior observed, it was established that this increase in energy did not provide any excessive breakage, but rather, improved the coalescence due to an improved liquid–solid distribution. However, since the nuclei formation occurred via the immersion nucleation mechanism, the eventual increase in the granule size/granule growth was due to increased layering-dominant growth. Layering-dominant growth granules lead to the formation of porous granules [44], and thence, we see an increase in the porosity of the granules with an increase in the screw speed from 300 rpm to 500 rpm. When the screw speed was increased to 700 rpm, the fill level of the granulator did not decrease notably, but the $E_{s}$ increased more than three times (Table 3). As discussed earlier, this increase in $E_{s}$ facilitated the breakage of the weak immersion nuclei and granules formed via layering-dominant growth, which resulted in the exposure of fresh surface wetted areas at an increased surface area-to-volume ratio of the granules. This essentially increased the liquid saturation of the granules, which eventually promoted granule growth. The agglomeration as a result of breakage could be due to aggregation instead of layering, which could be due to the increased extent of consolidation, leading to an increased surface area-to-volume ratio. Unlike the layering mechanism, the aggregation mechanism leads to the formation of dense and strong granules [2,44,51]. Hence, due to a shift in the growth mechanism, a decrease in the porosity of the granules was observed when the screw speed was increased to 700 rpm.

**Figure 4:**
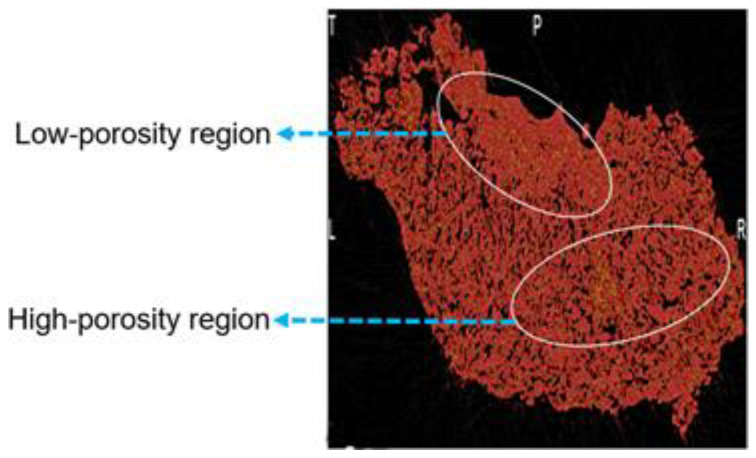
Microstructure investigation of the granule obtained at a screw speed of 300 rpm.

Furthermore, from close observation, a non-uniform distribution of porosity can be observed for granules formed at 300 rpm compared to the granules obtained at 500 rpm and 700 rpm. The top part of the granule has a high density/low porosity region, whereas in the bottom right corner, the density is low, and consequently, the porosity is high (Figure 4). At a screw speed of 300 rpm, the highest fill level and lowest $E_{s}$ value were observed (Table 3). Studies in the literature have stated that at higher fill levels, there could be localized zones of high compaction/shear forces [35], which could be the reason for the non-uniform porosity distribution. Another plausible reason for this observation is that at a screw speed of 300 rpm, the energy supplied to the powders is very low, and the mixing in the barrel of the twin-screw granulator is very poor; this could have resulted in a localized layering-dominant growth of granules without proper mixing, resulting in such a poor porosity distribution. Moreover, from Figure 2b,c, it can be observed that the granules obtained at a screw speed of 500 rpm have the most uniform pore network, and the granules obtained at a screw speed of 700 rpm have the greatest fraction of closed pore networks. These observations could be due to the difference in the granule-growth mechanisms observed at these different screw speeds.

Layering-dominant growth resulted in the highest porosity and the most uniform pore network, whereas the aggregation mechanism, due to breakage-dominant growth, resulted in the lowest porosity and a high volume of closed pores. Overall, a complex interplay between the fill level, $E_{s}$, and axial dispersion dictated the microstructure of the granules.

### 3.3. Effect of Kneading Elements

For this set of experiments, zone 4 was used as the granulation zone, the screw speed was set to 500 rpm, and an L/S ratio of 100% was used. An increase in the number of kneading elements results in a decreased conveying capacity due to a decreased free volume-to-maximum volume ratio, which eventually results in an increased MRT and holdup. Hence, the fill level of the twin-screw granulator barrel generally increases with an increase in the number of kneading elements. But from Table 3, it can be seen that when the number of kneading elements is increased from four to six, the MRT decreases, and thence, the fill level also decreases. This could have stemmed from a difference in the mixing and growth dynamics, which will be investigated in detail in this section.

**Figure 5:**
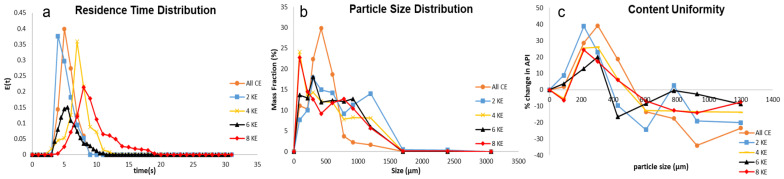
Effect of the number of kneading elements on the (**a**) residence time distribution, (**b**) particle size distribution, and (**c**) content uniformity.

Mixing in a fluid system occurs when particles/streams of different temporal histories encounter each other [4]. It can be observed from Figure 5a and Table 3 that with an increase in the number of kneading elements, the mixing in the system and the dispersion coefficient also increased. This can be attributed to the impact of the kneading elements on the flow pattern inside the twin-screw granulator barrel. The mechanism of action of the kneading elements on the flow pattern is breaking the smooth bulk flow and creating multiple smaller flow streams with complicated random motions [4,52,53]. The multiple smaller streams created, due to their random haphazard motions, increase the probability of particles/streams with different temporal histories encountering each other, leading to improved mixing. With an increase in the number of kneading elements in the kneading zone, the number of smaller streams with random flow patterns being created increases, which ultimately increases the dispersion coefficient and improves the mixing in the system [4].

**Figure 6:**
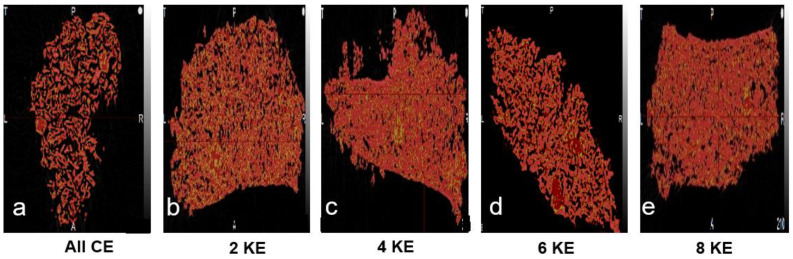
Effects of kneading elements on the granule microstructure: (**a**) 0 kneading elements/all conveying elements, (**b**) 2 kneading elements, (**c**) 4 kneading elements, (**d**) 6 kneading elements, and (**e**) 8 kneading elements.

From Figure 5b, it can be seen that the PSD of the granule is significantly affected by the kneading elements, and the trends obtained can be explained by the shifts in the granulation growth mechanisms as a function of the kneading elements, as discussed in the previous paragraph. When all conveying elements were used in the screw configuration, a unimodal curve with a narrow particle size distribution was obtained (Figure 5b). This is a result of poor mixing dynamics inside the barrel of the twin-screw granulator obtained with this screw configuration, which is reflected in the very low value of the axial dispersion coefficient (Table 3). As a result of the very poor mixing dynamics, the liquid–solid distribution in the barrel of the twin-screw granulator is also very poor in this condition. This consequently implies that the liquid is trapped within a few nuclei, and the growth of these few particles occurs via the layering of the finer particles onto the few liquid-rich nuclei. Due to poor axial mixing and liquid–solid distribution, the surface wetness of the particles is low, and the exposed wetted surfaces of the few nuclei also get saturated relatively quickly due to the layering of the finer particles. This leads to a low extent of granule growth, and due to a lack of sufficient energy (low $E_{s}$) provided to the powders, breakage of the particles is also almost non-existent in the system. Hence, we get a narrow unimodal PSD with only conveying elements for this type of formulation, which is typical of nucleation-dominant growth systems [38,39,54]. When a kneading zone with two kneading elements was added, the axial dispersion inside the twin-screw granulator barrel increased, which resulted in an improved liquid–solid distribution. This improvement in the liquid–solid distribution could be due to the breakage of the initial nuclei formed or the consolidation of the granules. However, due to the very small size of the kneading zone, the extent of liquid–solid distribution is stunted, and consequently, the improper and partial distribution of liquid occurs. This leads to the promotion of granule growth and the formation of large granules/agglomerates (>1000 μm) to an extent, and overall, due to the improper and partial liquid distribution, the granules will result in a wide particle-size distribution (Figure 6b).

With a further increase in the kneading elements, the axial dispersion and mixing efficiency of the system increased. As expected, this improved the liquid–solid distribution in the system further. From Figure 6b, it can be observed that an increased fine fraction was obtained when the screw configuration had been employed with four kneading elements. This could be due to the increased breakage occurring in the kneading zone. Essentially, the improvement in the liquid–solid distribution was facilitated by breakage in this case, which led to the formation of newer wetted surfaces. The increased fine formation led to the deterioration of the flowability of the granules in the system, which, in turn, increased the MRT (apart from the increase due to reduced free space in the granulator) and torque significantly. This increase in the torque eventually increased the energy supplied per unit time per unit mass ($E_{s}$), even though the holdup increased significantly due to the increase in the MRT ($E_{s}$ is inversely proportional to holdup). Even though the increase in $E_{s}$ between the two kneading elements and four kneading elements configurations is nominal, the average total energy ($E_{avg}$) imparted to a particle is significantly higher due to a significant increase in the MRT. The average total energy ($E_{avg}$) imparted to the particle (unit mass) is a product of $E_{s}$ and MRT and has the units of J/g. The $E_{avg}$ increased from 395.86 J/g to 617.29 J/g when the kneading elements were increased from two to four. Hence, significant breakage was observed at this screw configuration, which resulted in increased fine formation and a reduction in large granule formation (>1000 μm, some large granule formation occurred due to an increased liquid–solid distribution). This feature of increased $E_{s}$ at an increased holdup is unique to kneading elements; this is also the reason for the improved mixing efficiency with an increase in the kneading elements, as $E_{s}$ is directly proportional to the axial dispersion coefficient [4].

When the number of kneading elements was increased to six kneading elements, as expected, the axial dispersion increased, but the MRT of the system decreased despite the reduced free space in the twin-screw granulator barrel. This decrease in the MRT is due to the improved flowability of the powder–liquid system attained at this screw configuration. This improved flowability is, in turn, a result of the improved coalescence attained due to an increased axial dispersion and improved liquid–solid distribution. The improved flowability achieved also decreased the torque of the system, ultimately decreasing the $E_{s}$ applied to the material in the barrel. All these factors together decreased the average total energy imparted to a particle/material per unit mass ($E_{avg}$) from 617.29 J/g at four kneading elements to 458.36 J/g at six kneading elements. This reduced total energy imparted to the granules at six kneading elements decreased the breakage occurring in the granulator. Rather, at this energy level, the increased surface wetness is due to the increased extent of consolidation, which essentially helps improve the liquid–solid distribution and promotes granule growth. Hence, at six kneading elements, the improved coalescence growth led to the formation of uniform weight fractions of granules (Figure 6b).

When the number of kneading elements was increased to eight, the cycle of an increased MRT and increased $E_{s}$ occurred again, which ultimately increased the $E_{avg}$ (the average energy imparted to a particle) to 1217.77 J/g. This $E_{avg}$ value is greater than the one obtained at four kneading elements, and therefore, the extent of breakage is larger. From Figure 5b, it can be observed that a significant reduction in the granule formation in the size class between 1000–1400 μm occurred (compared to two- and four-kneading element cases). Ultimately, this resulted in a narrow bi-modal distribution, and the width of the PSD curve obtained was comparable to the six-kneading element case. The bimodality of the PSD at eight kneading elements is a result of the breakage, but the breakage also improved the liquid–solid distribution, resulting in the promotion of the growth of granules, especially for the granules between the sizes of 710 and 1000 μm. It was observed that at the six-kneading element configuration and eight-kneading element configuration, the formation of granules in the range of 710 and 1000 μm was the largest, and this could be due to the superior liquid–solid mixing obtained under these conditions.

Overall, it was observed that the growth of granules with an increase in the number of kneading elements altered between being breakage driven and layering driven. This was due to a complex interplay between the axial mixing (resulting in an improved liquid–solid distribution) and the average total energy imparted to the material per unit mass ($E_{avg}$).

An increase in the number of kneading elements results in an improved axial dispersion and liquid–solid mixing. This improved liquid–solid mixing results in an increased surface wetness and the promotion of granule growth and solid component exchange. This relationship between axial mixing and solid component exchange can be observed in Figure 5c, where the content uniformity of the granules improved with an increase in the number of kneading elements. This was also reflected in the de-mixing potential values obtained (Table 3), which decreased with an increase in the number of kneading elements. A slight increase in the de-mixing potential was observed when the kneading elements were increased from six to eight; this could be due to an increased fine-particle formation under these conditions. Since the calculation of de-mixing potential is directly proportional to the weight fractions, an increased fine-particle formation could have led to a higher value of de-mixing potential.

From Figure 6, it can be observed that the microstructure of the granules reflects the growth mechanism occurring at that particular screw configuration. The granule formed using conveying elements had a very high porosity (Figure 6a), as expected from the nucleation-type growth occurring under these conditions. At the two-kneading element screw configuration, the granules formed were fairly porous, and it was observed that the porosity of the granule was unevenly distributed, with a dense core at the bottom right corner and larger pores distributed in the other parts of the granule (Figure 6b). This is reflective of the type of growth occurring in the system under these conditions, which was partially breakage and partially layering due to improper mixing and the low total energy imparted. When the kneading elements were increased to four, the microstructure of the granule was denser, due to improved mixing and the large total energy imparted, which promoted growth via the coalescence of broken wet granules/nuclei. However, the porosity of the granule was found to be somewhat unevenly distributed (Figure 6c). The porosity of the granule increased again at six kneading elements due to a reduction in breakage at this setting. Moreover, the porosity was found to be well distributed across the granule. These observations could be due to an improved axial dispersion and liquid–solid distribution resulting in an improved uniform layering type growth, under these conditions. Finally, at eight kneading elements, the granule density was the highest, resulting in a very low porosity. This could have been due to the granule growth being promoted by increased breakage under these conditions. At such high energies, all weak wet agglomerates break down, resulting in the exposure of wet surfaces at a high surface area-to-volume ratio. These smaller surface wet granules also undergo coalescence under such high energy conditions, resulting in granules with very high densities.

### 3.4. Effect of Kneading Zone Position

**Figure 7:**
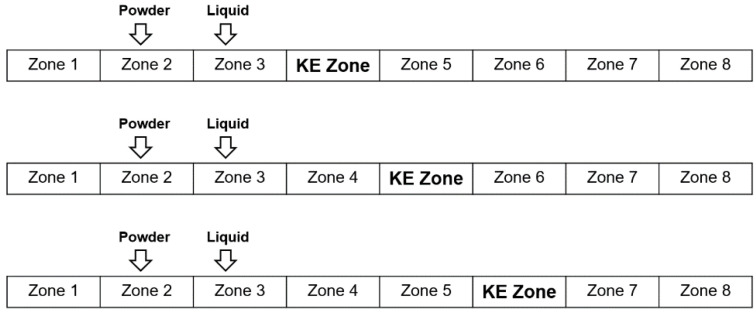
Positions of the kneading zone relative to the liquid addition zone used in this study.

For this set of experiments, a screw speed of 500 rpm was used, and six kneading elements were employed in the granulation zone. An L/S ratio of 80% was used for these experiments. In this part of the study, the position of the kneading zone was varied between zone 4, zone 5, and zone 6. This resulted in 5.5 cm (5 conveying elements), 11 cm (10 conveying elements), and 16.5 cm (15 conveying elements) of distance between the liquid addition zone and kneading zone (Figure 7) at each of the kneading zone positions, respectively.

The distance between the liquid addition zone and the kneading zone or the length of the post-liquid addition conveying zone determines the extent of nucleation occurring in the system, affecting the degree of nucleation-dominant growth in the twin-screw granulator. The wetting and nucleation process in the twin-screw granulator takes place in less than one or two seconds. Such a quick kinetic process, along with the dropwise addition of liquid binder, often results in some degree of non-uniformity in the distribution of the liquid binder across nuclei. This leads to the formation of larger wet nuclei/agglomerates and a dry fine material. This is generally alleviated by efficient solid–liquid mixing in the kneading zone through breakage and/or the consolidation-driven redistribution of the liquid and solid components. Suppose the distance between the liquid addition zone and kneading zone is increased. In that case, the initial segregation created in the liquid addition zone due to the formation of large wet nuclei/agglomerates and fine material gets aggravated, worsening the quality of the nuclei. The deterioration in the quality of the nuclei mainly causes a poor liquid distribution, trapping the liquid within a few agglomerates, which eventually will result in a poor solid distribution because of partial wetting and liquid entrapment.

**Figure 8:**
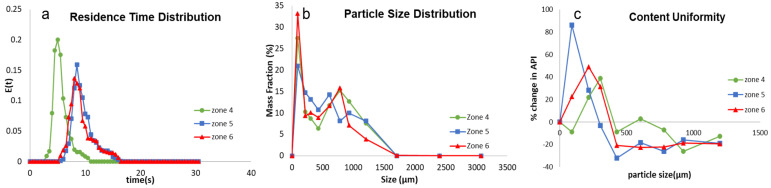
Effect of the kneading zone position on the (**a**) residence time distribution, (**b**) particle size distribution, and (**c**) content uniformity.

From Figure 8a, it can be seen that the axial dispersion and mixing initially decreased and then increased with the shift in the kneading zone position. This is most likely due to a change in the mechanism occurring in the kneading zone relative to the quality of the nuclei fed to it. When the kneading zone was at zone 4, the quality of the nuclei entering the kneading zone had the lowest degree of non-uniformity in terms of the liquid and solid component distribution. Hence, the kneading block has a relatively easier job of redistributing the solid and liquid components and improving the solid–liquid distribution. This improvement in distribution would have been achieved by consolidation or breakage, depending on the saturation state of the granule and the energy provided by the system. In this case, the L/S ratio used was 80%, which could lead to less surface liquid available on the granules. The average total energy imparted to a particle/material per unit mass ($E_{avg}$) when the kneading zone position was at zone 4 was equal to 745 J/g (Table 3), which is in the relatively higher range of $E_{avg}$. Hence, with less surface liquid and a relatively high $E_{avg}$, the combination could have led to partial consolidation and breakage. This improved liquid–solid distribution due to partial consolidation and breakage could have led to a layering-dominant growth, resulting in a sharp bi-component distribution, since the liquid saturation is already low (Figure 8b). As expected, this improved liquid–solid distribution at zone 4 also resulted in the best content uniformity of the granules (Figure 8c).

When the kneading zone position was moved to zone 6, the degree of non-uniformity of the nuclei fed to the kneading zone was the highest with respect to liquid binder encapsulation and solid component distribution. This meant the quality of the nuclei entering the kneading zone was very poor. In this case, the nuclei fed to the kneading zone would mostly have a few large nuclei/agglomerates entrapping most of the liquid. Since MCC is hygroscopic, these large nuclei will have a disproportionately high MCC content, leaving behind a large portion of unwetted fine particles of the API (APAP). Under these conditions, the kneading zone cannot effectively distribute the liquid and solid components, worsening the mixing (Figure 8a). In this case, the $E_{avg}$ was equal to 1118.06 J/g (Table 3), meaning the kneading zone acts as a complete breakage zone. This breakage does facilitate some liquid–solid distribution, but due to the very poor quality of the nuclei and low surface liquid of the granules at an 80% L/S ratio, sufficient liquid cannot be drawn out to promote the formation of large particles (>1000 μm). Instead, it can be observed from Figure 8b that the promotion of growth due to breakage, in this case, was mostly limited to granules between 500 to 850 μm. Due to excessive breakage, the excessive formation of fine particles is expected. Hence, the kneading zone at the zone 6 position resulted in a bi-modal particle size distribution, with a prominent peak in the finer particle region. The breakage mechanism in the kneading zone could have resulted in the breakage of MCC-rich granules into fine particles, ultimately aiding in the improvement in the content uniformity of the granules (Figure 8c).

When the kneading zone was moved to zone 5, the quality of the nuclei entering the kneading zone had an intermittent quality. The liquid binder encapsulation and solid component distribution would also have been between the two extremes discussed earlier. This makes the job of the kneading zone, in terms of the solid–liquid redistribution, relatively difficult, resulting in poor mixing. The $E_{avg}$ when the kneading zone position was at zone 5 was calculated to be equal to 1002.88 J/g, which would again indicate that the predominant mechanism in the kneading zone of the twin-screw granulator would be breakage. This breakage facilitates an increase in the surface wetness of the granules at a higher surface area-to-volume ratio, promoting granule growth. However, since the surface liquid available for granule growth is already low, the growth promoted will be via the layering-dominant mechanism. Hence, the initial breakage of the wet nuclei, followed by the layering-dominant growth of the broken granules, results in a multi-modal distribution (Figure 8b) in this case as well. As mentioned earlier, with an increase in the length of the distance between the liquid addition and kneading zone, segregation between wet nuclei/agglomerates and finer material increases. This deterioration of segregation is due to the difference in the flow properties of the wet agglomerates and the fine materials, causing poor mixing in the conveying zone, leading to partial encounters amongst particles (meaning wet agglomerates interact/mix with wet agglomerates and drier fines with drier fines) [53]. Due to these partial interactions, the initial inhomogeneity created in the liquid addition zone due to partial wetting worsens, creating the worst mixing scenario and forming the most non-uniform granules (Figure 8c). Since the available surface liquid on the granules when the kneading zone is situated in zone 5 compared to zone 6 is higher, the formation of larger particles (>1000 µm) was higher in the former case.

The D50 value decreased with the increase in the post-liquid conveying-zone length. This could be due to the increased breakage and reduced free surface liquid available when post-liquid addition conveying-zone length was increased. When only conveying elements were used in the screw configuration, it was observed that the maximum fraction of mass was obtained in the size fractions of 350 and 500 μm (Section 3.3). When investigating the effects of kneading zone positions, the granule growth was promoted beyond this point across all the positions of kneading zones (a dip in the PSD is observed at this size fraction), as shown in Figure 8b. Interestingly, the mass fraction obtained in this size class depended on the extent of mixing obtained at each kneading zone position.

The above results show that, in general, it is essential to keep the kneading zone as close to the liquid addition zone as possible to promote liquid–solid distribution, resulting in granule formation with a uniform content. This is especially true when the available free surface liquid for granule growth is low.

**Figure 9:**
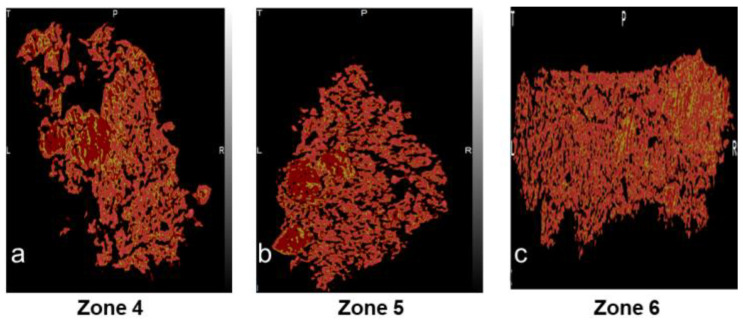
Effect of the kneading zone position on the granule microstructure in (**a**) zone 4, (**b**) zone 5, (**c**) zone 6.

Since the primary mechanism of growth was layering-dominant growth, irrespective of the kneading zone position, the granules formed across all three kneading zone positions were relatively porous (Figure 9). However, it can be observed that the pore distribution becomes non-uniform, with a rightward shift of the kneading zone position. Especially for the granules achieved at a kneading zone position of zone 6 (Figure 9c), a distinctive high-density region can be observed at the top right corner of the granule. This non-uniformity in the pore distribution could be due to increased breakage-facilitated layering-dominant growth with an increase in the post-liquid addition conveying-zone length. Furthermore, it was observed that the fraction of undispersed MCC-rich nuclei (${nuclei}_{UD}$, the method of calculation of this metric has been adopted from the authors’ previous paper [29]) increased from 22.4 to 35 with the shift in the kneading zone position from 4 to 6. This shows that the extent of liquid binder entrapment, which directly correlates to a lower extent of consolidation, affects the extent of memory retention of the nuclei within the granule.

## 4. Important Physically Relevant Intermediate Parameters of Mixing in TSG

The four mechanistically relevant intermediate parameters affecting mixing in TSG have been identified as the fill level, liquid saturation, the extent of nucleation, and powder wettability. The effect of these intermediate parameters on the mixing and granule growth dynamics will be discussed in detail in this section.

### 4.1. Fill Level

The fill level is known to affect the shear applied to powders [33,55,56]. By controlling the shear applied to the powders inside the twin-screw granulator, the fill level controls the number and frequency of the collisions occurring inside the twin-screw granulator. Since mixing occurs when streams/particles of different temporal histories encounter each other [4], the number of collisions and the collision frequency affect the mixing significantly. Hence, the fill level is an important parameter that affects the mixing and granule growth inside the twin-screw granulator. The two of the most critical parameters that have a distinctive effect on the fill level in the twin-screw granulator are the number of kneading elements and the screw speed [4,8,27,57].

As explained in the earlier section, an increase in the screw speed leads to an improvement in the mixing dynamics of the system. This improvement in mixing results in an improved solid–liquid distribution, either through an increase in the extent of consolidation or an increase in the breakage. The improvement in the solid–liquid distribution leads to an increase in the surface wet area, which promotes granule growth and solid component exchange. The growth can be either via the coalescence between two surface wet granules or by the layering of smaller, finer particles onto the larger surface-wet granule.

Similar to the screw speed, an increase in the number of kneading elements also leads to an improvement in mixing, as observed in the previous section. Due to improved mixing dynamics, an increase in the number of kneading elements leads to an improvement in the solid–liquid distribution, which eventually results in granule growth. As explained in the earlier section, this improvement in the solid–liquid distribution could be due to an increase in the extent of consolidation or an increase in the breakage depending on the fill level and energy supplied to the powders ($E_{s}$). The type of granule growth can either be layering dominant or viscous dominant, depending on the type of nucleation mechanism. Moreover, an increase in the kneading elements can switch the growth mechanism from layering dominant to aggregation dominant, depending on the availability of free liquid on the granule and the surface area-to-volume ratio of the granule. The increase in the surface area-to-volume ratio due to the breakage of particles can lead to aggregation-dominant growth, either due to an increase in viscous dissipation or deformation.

### 4.2. Liquid Saturation of Granules

Liquid saturation is defined as the ratio of liquid binder present in a granule to that of the granule void space, which is essentially the fraction of granule porosity occupied by the liquid binder [58]. Liquid saturation affects the kind of growth that the particles undergo in a system; at a high liquid saturation due to increased viscous dissipation, coalescence growth is promoted immediately [46]. On the contrary, at a low liquid saturation, the promotion of granule growth via coalescence depends on the extent of consolidation and the consolidation rate of the granules [46,51]. The consolidation of the granules essentially ensures that the particles in the granule are rearranged/compacted to squeeze the binder liquid into the exterior surface of the granule [2]. Consolidation itself is affected by various parameters such as the shear applied to the system, primary particle size, primary particle compressibility, binder viscosity, etc., apart from the state of liquid saturation itself [47,48].

**Figure 10:**
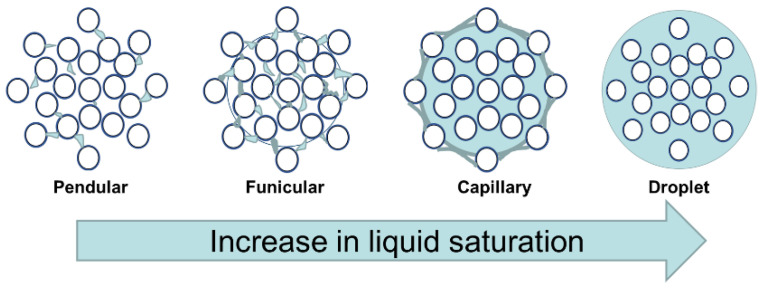
Different stages of liquid saturation in a granule [60].

One of the most fundamental parameters affecting liquid saturation is the quantity of binder liquid in the system, which, in turn, is governed by the liquid-to-solid (L/S) ratio. With an increase in the L/S ratio, the liquid saturation of the granules increases, which moves the granule from a pendular system to a droplet system, as shown in Figure 10. With an initial increase in the L/S ratio, the interparticle friction within the granule decreases due to an increase in lubrication, which, in turn, promotes consolidation due to deformation [51]. With a further increase in the L/S ratio, the effect of liquid saturation on interparticulate friction decreases, and its effect on viscous dissipation increases (at this stage, the extent of consolidation decreases), which ultimately increases the probability of Type 1 coalescence [2,51,59].

The collision between two approaching surface wet granules results in a successful coalescence if the kinetic energy of the collision is completely dissipated during the collision [46]. Many collisions occur inside a granulator, of which only a fraction of collisions result in successful coalescence and granule formation. Yet, there is a very important mechanism occurring during unsuccessful collisions, which is the liquid transfer between granules during the separation stage of the collision. This process results in liquid redistribution amongst the colliding granules, which determines the eventual growth of these granules, and hence, in general, the granule growth occurring in the system. The nature of this redistribution is highly dependent on the liquid saturation state of the colliding granules. This process of liquid distribution due to collisions is nothing but an act of mixing, and it is very important to understand these dynamics in a fast-paced system like TSG. For example, if a collision occurs between two low-saturation granules, the collision might not result in any redistribution, or due to the impact, there might be some liquid squeezed in the contact zone of the granules (still insufficient for successful coalescence). The squeezed liquid could be disproportionately distributed amongst the colliding granules depending on the characteristics of the colliding granules, such as the size, porosity, etc. Similarly, the distribution of the liquid between two high-saturated granules could eventually lead to the growth of one granule and stagnation in the growth of the other.

The kneading elements and screw speed also affect the liquid saturation of the granules through solid–liquid distribution. As mentioned earlier, the enhanced mixing in the system achieved with an increase in the screw speed or number of kneading elements leads to an improved solid–liquid distribution. This improved liquid–solid distribution is obtained either due to the enhanced extent of consolidation or breakage of granules, leading to the exposure of new wet surfaces.

### 4.3. Extent of Nucleation

Unlike a batch high-shear granulator, continuous twin-screw granulator is regimen-separated [4,8,34], i.e., the granulation rate mechanisms occur sequentially in the twin-screw granulator (both in space and time). This enables the increased control of the granulation process in the twin-screw granulator [4]. Depending on the screw configuration, the consolidation and growth and the breakage and attrition regimens can coincide within the same kneading zone, making it a cyclic process (breakage promotes liquid distribution, which further promotes growth; hence, cyclic) rather than a sequential process. However, the wetting and nucleation mechanism is usually regimen separated, and it generally occurs in a liquid addition zone prior to the kneading zone. The addition of the binder liquid to the kneading zone can lead to a periodic surge in the output at the exit of the granulator, which implies a periodic unsteady-state operation, leading to poor granule quality [53,61]. Hence, the liquid addition zone is most commonly comprised of the conveying elements. The granules formed in the wet granulation process often retain a “memory” of the constituent nuclei, and thus, the wetting and nucleation process critically affects the granule quality attributes of the resultant granules, such as the size, microstructure, etc. [62,63].

It was seen in an earlier study, that the nucleation kinetics in the liquid addition zone affect the mixing dynamics, and thus, the granule growth dynamics occurring in the kneading zone of the twin-screw granulator, by affecting the quality of the nuclei entering the kneading zone [29]. One of the key parameters that represents the nucleation kinetics in a twin-screw granulator is the extent of nucleation. The extent of nucleation affects the quality of the nuclei entering the kneading zone and consequently influences the dynamics of granule formation. The extent of nucleation is affected by two key factors: the binder viscosity and the distance between the liquid addition zone and the conveying zone. In an earlier study, it was shown that the binder viscosity is the primary factor affecting the ratio between the drop penetration time and encounter time ($D_{t}/E_{t}$), which determines the extent of nucleation occurring in the process. If the drop penetration time is lower than or approximately equal to the encounter time ($D_{t}/E_{t}\leq 1$), the nucleation process is completed, and the properties of the nuclei formed depend on the hydrophobicity of the primary powders and the blend uniformity of the primary powders in the liquid addition zone. Since the common practice is to pre-mix the primary powders before the TSG process, and generally, the liquid addition zone is right next to the powder addition zone, it is safe to assume that the blend uniformity of the powders in the liquid addition zone is uniform. Hence, the primary factor affecting the nuclei properties, in the case of $D_{t}/E_{t}\leq 1$, would be the hydrophobicity of the blend. But when the drop penetration times are larger than the encounter time ($D_{t}/E_{t}>1$), the nucleation process is not completed, because a significant part of the binder droplet remains on the powder surface during the encounter with a different set of powders at the intermeshing section. This excess free liquid present on the powder surface is inhomogeneously engulfed by powders at the intermeshing zone, leading to the formation of inhomogeneous nuclei with locally trapped liquid. This essentially concludes the existence of excess free binder liquid being available locally at higher liquid-binder viscosity levels. Essentially, in the case of $D_{t}/E_{t}>1$, the nucleation process is incomplete, leading to the formation of inhomogeneous nuclei. With the increase in the binder viscosity, the drop penetration time of the liquid droplet increases, switching the nucleation from a complete nucleation process to an incomplete, inhomogeneous one. The degree of inhomogeneity of the nuclei formed increases with the binder viscosity.

The second parameter, the distance between the liquid addition zone and the kneading zone or length of the post-liquid addition conveying zone, determines the degree of nucleation-dominant growth in TSG. Generally, the wetting and nucleation process in TSG takes place in less than 1 or 2 s, and such a quick kinetic process along with the dropwise addition of liquid binder often results in a non-uniform distribution of the liquid binder, leading to the formation of larger wet nuclei/agglomerates and dry fine material [49,53,61,64,65]. This is generally alleviated by efficient liquid–solid mixing in the kneading zone, either through breakage or the consolidation-driven redistribution of the liquid [32,49]. If the distance between the liquid addition zone and kneading zone is increased, the initial segregation created in the liquid addition zone due to the formation of large wet nuclei/agglomerates and fine material gets aggravated. This deterioration of segregation is caused by the difference in the flow property of the wet agglomerates and the fine materials and poor mixing in the conveying zone, leading to partial encounters (meaning wet agglomerates interact/mix with wet agglomerates and drier fines with drier fines) [53]. This traps the liquid poorly within a few large agglomerates, making it difficult for the kneading zone to carry out efficient solid–liquid mixing. This ultimately affects the growth dynamics and quality of the resultant granules formed, such as excessive fine formation, etc.

### 4.4. Powder Wettability

Typically, in TSG, if the liquid binder droplet size is considerably larger than the powder, under such circumstances, the nuclei formed in the twin-screw granulator undergo either the immersion nucleation mechanism or solid spreading nucleation mechanism. The type of nucleation mechanism that the powder–liquid system undergoes is dependent on the contact angle of the powder mix with the liquid binder and the spreading coefficient of the liquid binder over the solid powders [2]. Immersion nucleation occurs when the liquid phase spreads over the solid phase; during immersion nucleation, the liquid phase completely penetrates the powder bed and forms nuclei with saturated pores [2]. On the contrary, solid spreading nucleation typically leads to the formation of highly porous nuclei, and during this process, the solid particles spread over the liquid surface to form the nuclei [66,67]. Immersion nucleation results in the formation of denser nuclei that are slightly larger than the size of the liquid droplet, whereas solid spreading nucleation leads to the formation of porous nuclei, and the size of the nuclei is roughly similar to that of the liquid droplet added. The hydrophobicity of the powder mix is the key factor that affects the nucleation mechanism in a system; hydrophilic powders tend to undergo immersion nucleation, whereas hydrophobic powders undergo solid spreading nucleation [68].

The type of nuclei formed dictates the mixing dynamics and the resultant growth dynamics of a TSG system. Even though the pores of the immersion nuclei are saturated, the amount of surface liquid present on the nuclei is still pretty low, leading to its characteristic brittle nature. In the kneading zone of the twin-screw granulator, at a low shear/collision velocity, the immersion nuclei can promote mixing due to the improvement in the liquid–solid distribution, which consequently promotes the growth of the particles. At a high shear/collision velocity, the kneading zone can promote the breakage of these nuclei, which can lead to a promotion or deterioration of the mixing, depending on the saturation and surface-to-volume ratio of the resultant broken granules.

Solid-spread nuclei or nuclei formed due to hydrophobic powders mimic the properties of the entrapped liquid and are highly deformable [66]. Planchette et al. [69] studied the collision of solid-spread nuclei, also known as liquid marbles [70], onto a solid surface and found that there can be three consequences of this collision: (a) rebound, (b) transition non-bouncing, and (c) rupture. Due to the restrictive space in a twin-screw granulator and the high energy supplied to the powders, it is fair to assume the most common scenario for the collision of solid spread nuclei is rupture. But in the minority of the cases where the non-bouncing of these nuclei occurs (low shear environment due to very low or no kneading elements and a low screw speed), these nuclei will collide with other granules and consequently lead to the surface growth of granules [44]. But in the more common scenario, the rupture of the nuclei will lead to an increase in the local availability of the liquid, and depending on the location of the rupture (conveying or kneading zone), this can either promote or deteriorate mixing, consequently leading to improved coalescence or an increased extent of liquid encapsulation, respectively.

Since the nucleation times are in the order of one or two seconds, the occurrence of solid spread nucleation in TSG is rare (solid-spread nuclei formation in general is a slow process). The more common scenario is the incomplete nucleation discussed in Section 4.3.

### 4.5. Mechanistic Process Map of the Physics Occurring Inside the Twin-Screw Granulator

**Figure 11:**
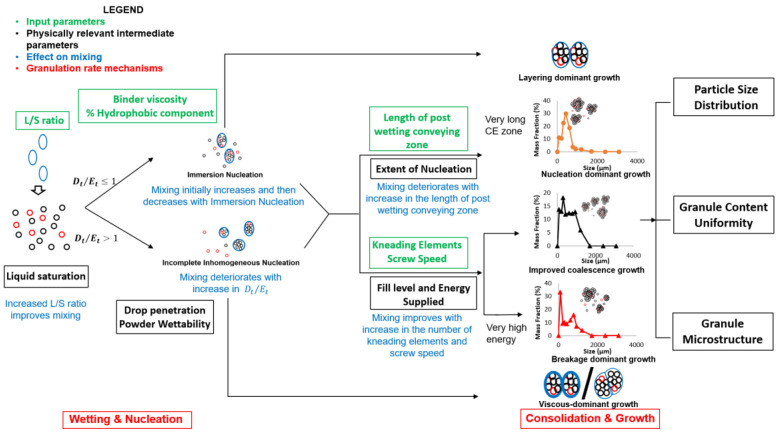
Mechanistic process map depicting the effects of input parameters on granule quality attributes through mixing and granulation growth mechanisms (red circles represent API particles, black circles represent excipient particles ad blue line over particle clusters represents surface water layer).

Based on the analysis conducted in this study, a mechanistic process map, as shown in Figure 11, was developed. This process map depicts the effect of mixing on the time-and-space evolution of the materials inside the twin-screw granulator, from liquid addition to granule formation. Once the liquid comes in contact with powders, depending on the ratio of the drop penetration time to encounter time ($D_{t}/E_{t}$), nuclei are formed via either complete immersion nucleation or incomplete inhomogeneous nucleation. Mixing gets affected based on the nucleation, and in turn, affects the granule growth mechanism. At their extremes, granules either grow via the layering-dominant mechanism or viscous-dominant growth mechanism. In between these two extremes, the granulation growth can switch between nucleation-dominant growth, improved coalescence, and breakage dominated depending on the extent of the nucleation, fill level, and energy supplied to the system. The type of granule growth mechanism finally determines the quality of the granules obtained in terms of the PSD, content uniformity, and microstructure. For example, the extreme layering-dominant growth results in a multi-modal PSD with a high fine fraction production, the homogeneity of the content is fairly uniform across size classes, and the resultant granules are very porous. On the contrary, viscous-dependent growth results in the formation of large dense granules with a bi-modal PSD (one of the peaks around large granules (>1000 μm)), and the granules formed will contain super potent fines. These properties can be adjusted by targeting a growth mechanism that is intermediate to these two extremes. For example, improved coalescence (attained by a sufficient improvement in consolidation) leads to the formation of granules with a uniform mass and size distribution and a comparatively narrow PSD, the homogeneity of the resultant granules is fairly good, and the resultant granules will have a high porosity. On the contrary, breakage-dominant growth will result in granules with a high porosity and bi-modal distributions. The developed process map can be adapted to soluble formulations as well, where the granule growth will predominantly occur between improved coalescence and breakage dominant at $D_{t}/E_{t}\;\leq 1$.

## 5. Conclusions

In recent years, studies have illustrated that to achieve a mechanistic understanding of the twin-screw granulation process, physically relevant mechanistic parameters have to be identified, and their effects on the granule quality must be analyzed to understand their mechanisms of action. Some studies have shown that mixing has a significant influence on the granule and tablet attributes in TSG. Therefore, in this study, the aim was to further the understanding of the effect of mixing on granule quality attributes. To do so, physically relevant intermediate parameters were identified and quantified, and their effects on mixing in TSG were established. Furthermore, using these parameters, the effect of mixing on granule attributes such as the PSD, content uniformity, and microstructure were analyzed. A new geometrical model to estimate the fill level in the twin-screw granulator was developed and validated against historical data present in the literature. New metrics to better estimate the energy provided by the system per unit mass and unit time ($E_{s}$) and the total average energy provided to a particle/unit material in the barrel ($E_{avg}$) have been introduced. Three parameters (fill level, $E_{s}$, and $E_{avg}$) were identified as the key physically relevant intermediate parameters. For an insoluble system such as the one used in this study, the relevant granulation growth mechanisms were identified to be layering-dominant growth, nucleation-dominant growth, improved coalescence growth, breakage-dominant growth, and viscous-dominant growth. The occurrence of the type of growth and its effect on the granule quality was dependent on mixing, and in turn, on the physically relevant intermediate parameters. In general, with an increase in axial dispersion and mixing, the solid–liquid distribution of the granules improved, which promoted granule growth. An improvement in axial mixing resulted in a better content uniformity of the granules, and the microstructure of the granules reflected the granulation growth mechanism occurring in the system. Furthermore, fundamental parameters affecting mixing were identified and theorized. The fill level, granule liquid saturation, extent of nucleation, and powder wettability were found to be the fundamental parameters that affect the axial dispersion and mixing inside the twin-screw granulator. The key input parameters affecting the fundamental parameters were identified and found to be the screw speed, kneading elements, liquid-to-solid ratio, binder viscosity, length of the post-wetting conveying zone, and hydrophobicity of the blend. Finally, a mechanistic process map was developed to depict the effect of mixing on the time-and-space evolution of the materials inside the twin-screw granulator from liquid addition to granule formation. This map helped explain the effects of physically relevant intermediate parameters on the granule quality attributes through mixing. The authors realize that to develop a complete map, an in-depth analysis similar to this study needs to be performed with a soluble formulation as well.

## Figures and Tables

**Table 1:** Material characterization of the chosen powders.

Material	D10 (µm)	D50 (µm)	D90 (µm)	Bulk Density (g/mL)	Cohesion (kPa)	Compressibility (%)	Permeability (cm^2^) (×10^−7^)
Dense APAP	40.67	178.9	380.9	0.48	0.328	15.88	6.369
Avicel PH 102	31.21	125.4	255.4	0.35	0.207	12.04	8.148

**Table 2:** Variation range of input factors investigated in this study.

Screw Speed (rpm)	300, 500, 700
Kneading elements (KE, number)	0, 2, 4, 6, 8
Position of KE zone	4, 5, 6

**Table 3:** Table describing the values of the calculated parameters across all the experiments.

L/S (%)	Screw Speed (rpm)	Kneading Elements (KE, Number)	Position of KE Zone	Axial Dispersion Coefficient, Da (cm^2^/s)	MRT (s)	DP (%)	Fill Level	Es (J/gs)	Eavg (J/g)
100	300	6	4	0.0504	13.15	19.79	0.053	49.7	653.56
100	500	6	4	0.6997	6.15	11.98	0.025	74.53	458.36
100	700	6	4	1.4486	5.15	7.77	0.021	233.83	1204.22
100	500	8	4	1.1922	10.91	13.63	0.043	111.62	1217.77
100	500	4	4	0.3506	7.39	16.77	0.031	83.53	617.29
100	500	2	4	0.1467	4.96	20.93	0.021	79.81	395.86
100	500	0	4	0.0223	4.77	24.75	0.021	73.53	350.74
80	500	6	4	0.5225	5.2	17.7	0.021	143.27	745
80	500	6	5	0.3474	9.33	33.46	0.038	107.49	1002.88
80	500	6	6	0.4124	9.19	45.97	0.037	121.66	1118.06

## Data Availability

All relevant data are presented in the article.
